# Anemia and blood transfusions in myelofibrosis: economic and organizational impact on Italian patients, caregivers and hospitals

**DOI:** 10.3389/fonc.2025.1549023

**Published:** 2025-03-07

**Authors:** Francesca Palandri, Alessandro Inzoli, Antonella Barone, Daniela Dordoni, Elisa Formenti, Giorgio Corradini D’Elia, Victoria Lucia Rabsiun Aramburu, Giuseppe Alberto Palumbo, Massimo Breccia

**Affiliations:** ^1^ Istituto di Ricovero e Cura a Carattere Scientifico Azienda Ospedaliero-Universitaria di Bologna, Istituto di Ematologia “Seràgnoli”, Bologna, Italy; ^2^ Hematology Unit, Ospedale Maggiore Azienda Socio Sanitaria Territoriale, Crema, Italy; ^3^ Associazione Italiana Pazienti Malattie Mieloproliferative (AIPAMM Organizzazione di Volontariato), Pavia, Italy; ^4^ GSK, Verona, Italy; ^5^ Life Sciences Division, Business Integration Partners S.p.A., Milan, Italy; ^6^ Dipartimento di Scienze Mediche, Chirurgiche e Tecnologie Avanzate “G.F. Ingrassia”, University of Catania, Catania, Italy; ^7^ Hematology, Department of Precision and Translational Medicine, Policlinico Umberto I, Sapienza University, Rome, Italy

**Keywords:** myelofibrosis, anemia burden, transfusion dependency, economic burden of disease, patient perspective, Italy

## Abstract

**Introduction:**

Anemia management in myelofibrosis (MF) remains a major challenge, often resulting in blood transfusions as the condition progresses. The BEAT project aimed to quantify the economic and organizational burden of anemia and transfusions in MF patients in Italy from the patient and hospital perspectives.

**Methods:**

Data were collected from two primary sources: (i) semi-structured interviews with 13 hematologists and 1 transfusionist from 13 Italian MF reference centers; (ii) an online questionnaire completed by 191 patients distributed by AIPAMM (Italian Association of Patients with Myeloproliferative Diseases). Patients were categorized into 9 patient types based on the Dynamic International Prognostic Scoring System (DIPSS), anemia status, and need for transfusions. The collected data was used to feed an analytical model to quantify time and costs for patients, caregivers and the healthcare system over one year for managing MF, MF-related anemia, and transfusion care for each patient type.

**Results:**

Transfusion dependent patients spend, on average, six times more time on MF care compared to non-anemic patients (133.1 vs 20.9 hours/year). Transfusion-related hospital visits represent a major burden, with waiting times accounting for 44% of total access time (about 7.3 hours). Annual hospital management time and estimated costs per patient are 17.0 vs. 5.2 vs. 3.5 hours/year, and €6,603 vs. €249 vs. €165/year for transfusion dependent, anemic non-transfusion dependent, and non-anemic patients, respectively. Indirect social costs for transfusion dependent patients (€2,332) are estimated to be six times greater than those for non-anemic patients (€367). Patient surveys confirmed the significant impact of transfusion dependency on work, social, and daily life, with scores of 4.5/5 for work and over 4/5 for social and daily life.

**Discussion:**

These findings highlight the urgent need for optimizing MF-related anemia and transfusion management to help mitigate the economic strain on healthcare systems and lessen the time-related and emotional impact on patients and caregivers.

## 1 Introduction

Myelofibrosis (MF) is a rare hematological neoplasia characterized by the abnormal proliferation of clonal hematopoietic stem cells, leading to progressive marrow fibrosis and disrupted blood cell production (1). It is the most severe and least common amongst the Philadelphia-chromosome-negative myeloproliferative neoplasms (MPNs), with an incidence of 0.3 to 0.8 per 100,000 individuals globally (2) and approximately 350 new diagnoses annually in Italy (3). MF primarily affects older adults, with a median age at diagnosis of 65 years old. However, around 25% of patients are diagnosed before the age of 56, and about 11% before age 46 (4). According to the 2022 classification by the World Health Organization (5) and the International Consensus Classification (6), MF can be divided into primary myelofibrosis, which arises *de novo*, and secondary myelofibrosis, which evolves from other types of MPNs, specifically post-polycythemia vera (post-PV MF) and post-thrombocythemia (post-ET MF).

Compared to other MPNs, MF is associated with a more debilitating disease course, poorer prognosis, and a higher risk of transformation to acute myeloid leukemia, which affects nearly 20% of MF patients (7, 8). Clinically, MF presents with a wide spectrum of signs and symptoms. Patients often suffer from debilitating constitutional symptoms such as fatigue, night sweats and fever, cachexia, itching, musculoskeletal pain, abdominal discomfort, early satiety, and perceived cognitive impairment (‘mental fog’). Other key clinical features include bone marrow fibrosis, splenomegaly and cytopenia (9). Anemia has a significant impact on patient quality of life (QoL) and serves as a critical indicator of disease severity and prognosis (8, 10, 11). In MF, anemia ranges from mild to severe and can lead to red blood cells (RBC) transfusion dependency in advanced stages. Around 40% of MF patients have hemoglobin (Hb) levels < 10 g/dl at diagnosis, and about 25% already require regular RBC transfusions (12).

Currently, therapeutic options for managing anemia in MF – mostly erythropoiesis-stimulating agents (13) and androgens such as danazol (14) – are limited and with minimal efficacy, especially for severe anemia (11). Consequently, RBC transfusions remain the primary intervention for severe anemia. However, frequent RBC transfusions carry health risks (i.e. iron overload and adverse reactions) and place a significant burden on patients, caregivers and the National Healthcare System (NHS) (11).

The critical role of anemia in determining MF prognosis is underscored by its inclusion in key prognostic models, such as the Dynamic International Prognostic Scoring System (DIPSS) (15), which evaluates risk based on anemia (Hb <10 g/dL), age >65 years, constitutional symptoms, leukocyte count >25 x 10^9/L, and peripheral blood blasts ≥1%. Each factor contributes to a cumulative score, stratifying patients into four risk categories: low, intermediate-I, intermediate-II, or high risk (15). Higher scores correspond to worse prognosis, with a reduced survival rate (less than 2 years for the high risk) (12).

The JAK-STAT signaling pathway plays a central role in MF pathogenesis. Like PV and ET, MF is characterized by the hyperactivation of this pathway, resulting in uncontrolled hematopoietic cell proliferation, elevated inflammatory cytokines production and excessive fibrotic tissue formation in the bone marrow. The JAK2 V617F gain-of-function somatic mutation is the most prevalent driver mutation in MF (16), with mutations in *CALR* (Calreticulin) and *MPL* (Thrombopoietin Receptor) also contributing to JAK-STAT signaling hyperactivation (17).

Following the discovery of JAK-STAT signaling involvement in MF pathogenesis, JAK-inhibitors particularly ruxolitinib and fedratinib have become widely used, providing significant symptomatic relief and reduction of the splenomegaly (18, 19). However, they do not alter the disease course or prevent progression. At present, allogeneic hematopoietic stem cell transplantation remains the only potentially curative treatment, yet its use is limited to a very small subset of eligible patients due to associated morbidity and mortality (20). Therefore, treatment of MF remains challenging and largely focused on alleviating symptoms. Additionally, due to the essential role of JAK-STAT signaling for physiological hematopoiesis, the use of ruxolitinib and fedratinib is associated with on-target hematological toxicity, leading to cytopenia, particularly anemia and thrombocytopenia (18, 21, 22). Thus, the use of JAK inhibitors can induce or exacerbate existing anemia in patients with MF, further complicating MF management.

Despite several studies showing the impact of MF on patient QoL (23–26), including the Italian ‘Back to Life’ project (27) which aimed to quantify the physical, emotional and financial impact of MF on patients and caregivers, no studies to date, at the best of the authors’ knowledge, have investigated the specific direct and indirect costs associated with MF-related anemia management and particularly RBC transfusion dependency in Italy.

The ‘Mapping the Burden in hEmatology for Anemic and Transfusion dependent patients with myelofibrosis’ (BEAT) project was designed to quantify the multifaceted burden of MF-related anemia and RBC transfusions, including patient and healthcare provider time, direct NHS costs, and indirect social costs for patients and caregivers. For that, we used quantitative data collected through semi-structured interviews to clinicians with experience in MF-management across Italian centers, alongside data on patient and caregiver perspectives gathered from an online survey, which was co-created in collaboration with AIPAMM (Italian Association of Patients with Myeloproliferative Diseases). Here, we first present key findings from our model analysis, integrating clinicians’ quantitative data and literature research. Then, we complement these results with insights from patients and caregivers (i.e. family members and close ones) to provide a more comprehensive assessment.

## 2 Methods

### 2.1 Data sources

The project collected data from three primary sources over the period between March 14^th^, 2024, and June 10^th^, 2024. Data were gathered from both patient and hospital perspectives to comprehensively evaluate MF impact in Italy.

- **Semi-structured interviews with clinicians:** Real-time semi-structured online interviews (see **Supplementary Material A**) were conducted individually with 13 hematologists and 1 transfusionist who manage MF patients from 13 Italian reference centers for myeloproliferative diseases. The clinicians were selected through convenience sampling based on their willingness to participate and expertise in MF. They responded based on their clinical experience and provided aggregate data based on hypothetical patient types, representing typical MF cases managed within their centers, without reference to specific patients. Clinicians were compensated for approximately 2 hours of their time at a rate in line with fair market value. Geographically, the clinicians who participated in the questionnaire provided representation across the entire national territory, with 62% based in northern Italy, 15% in central Italy, and 23% in southern Italy. Collectively, the centers where the clinicians practiced treated a total of 1,364 MF patients annually. Of these, 4 centers were classified as high-volume (treating more than 100 MF patients annually), while the remaining 9 centers were categorized as low-volume.
- **Patient questionnaire:** An online questionnaire (see **Supplementary Material B**) was specifically co-designed with AIPAMM for the project and distributed through its mailing list (227 contacts) and private Facebook group (891 members). The questionnaire was addressed to patients and caregivers who responded on their behalf. Participation was voluntary, with no selection of specific patients, and accessible only after providing written informed consent, in compliance with Italy’s Privacy Law [D.Lgs. 196/2003 and EU Regulation 2016/679]. A total of 191 responses were received. No identifiable personal data, including IP addresses, were collected, ensuring participants' anonymity and compliance with data privacy regulations. Additionally, no clinical data were collected. The data collection method did not require the project to have ethics approval.
- **Literature**: Additional cost data, including transfusion blood bags, personnel, and overheads costs, were sourced from the literature to complete the economic analysis.

The project complied with the EU General Data Protection Regulation [EU Regulation 2016/679] and was conducted in accordance with the ESOMAR (European Society for Opinion and Market Research) code of conduct and with the principles of the Declaration of Helsinki.

### 2.2 Patient types and identification of the patients’ pathway

Nine “patient types” were identified based on DIPSS risk, anemia status, and transfusion dependency to model the varying organizational and economic impacts associated with MF. Specifically, patients were categorized into three risk levels according to the DIPSS classification (low, intermediate I, and intermediate II/high). Within each risk level, patients were further stratified into three anemia-related categories, those who were not anemic, those who were anemic but did not require transfusions, and those who were anemic and required transfusions (see **Table 1**).

**Table 1:** Stratification of questionnaire respondents based on their self-reported DIPSS Risk and their clinical status.

Clinical Status	Self-reported DIPSS Risk level		
	Low (60)	Intermediate (69)	High (22)
No anemic	60% (36)	37% (26)	14% (3)
Anemic, without need for transfusion	38% (23)	41% (28)	36% (8)
Anemic, need for transfusion	2% (1)	22% (15)	50% (11)

Out of the 151 MF total patients who answered the three questions (their DIPSS risk, whether they suffer anemia and if they received at least one transfusion in the past year) with a conclusive answer and didn’t answer “I don't know/I don't want to answer” to either question, which were excluded.

DIPSS Dynamic International Prognostic Scoring System

To manage their MF and its associated anemia, patients follow four main types of contacts with the NHS, with the frequency of these accesses varying depending on which of the nine patient types they belong to.

1. *MF check-up:* Routine check-up visits to assess MF progression and anemia levels. Blood tests are typically (90% of cases) conducted in an external lab days before, to minimize in-hospital waiting times.
2. *Combined check-up and transfusion*: These accesses combine routine monitoring visits including assessment for transfusion eligibility, with transfusion administration in one access to streamline care. Blood tests could be conducted on the same day at the hospital (68% of cases) or the day before (32% of cases).
3. *Transfusion-only:* Focused on administering transfusion after a preparatory visit to ensure eligibility. Blood tests could be conducted on the same day at the hospital (68% of cases) or the day before (32% of cases).
4. *Blood test only:* Additional check-up blood tests performed at laboratories usually near the patient’s residence, with results sent to the treating physician for remote evaluation.

Based on input from clinicians during the interviews, we determined the frequency of each type of access for all nine patient types over a one-year period.

### 2.3 The average center

Data from clinicians across the 13 centers, distributed across Italy, were aggregated to create a hypothetical “average center”. The ‘average center’ model was constructed using a combination of weighted averages, simple averages, and estimates, as described here. Weighted averages were employed to provide unbiased estimates of descriptive statistics and ensure that the model accurately reflected the contributions of both high-volume centers (treating >100 patients annually) and low-volume centers (treating ≤100 patients annually). This approach allowed for statistically representative, population-based findings. A detailed explanation of the rationale and formula used to calculate weighted averages is presented in **Supplementary Figure 1**. Most patient-related parameters, such as the percentage of patients by clinical status or access type, were calculated using this method.

Conversely, parameters related to the organization of care (e.g., personnel time, waiting times, and activity times for patients) were determined by averaging the values reported by clinicians. For other parameters, such as the average number of caregivers per patient and travel times to the hospital, data were derived directly from patient questionnaire responses, as these were deemed the most reliable source of information for these metrics.

Finally, certain cost-related parameters, such as healthcare personnel costs and structural costs, were sourced from existing literature or publicly available datasets. A comprehensive list detailing the data sources and calculation methods for the parameters used to define the ‘average center’ is provided in **Supplementary Table 1**.

### 2.4 Outcomes

The BEAT project primarily assessed the economic and organizational impacts of MF in four dimensions: patient times, personnel times, direct hospital costs, and indirect costs on patients and caregivers. An analytical model for total cost analysis was developed and was fed by integrating aggregated data primarily from clinician interviews and, in specific cases, supplemented with patient questionnaire responses and literature data. The model mapped patient care pathways and allocated time and costs across the nine different patient types, guided by the frequency of specific access types identified during clinician interviews. It provided a framework to systematically estimate the organizational and economic burden of MF-related anemia and transfusions across three levels of aggregation: single access, annual MF management for a single average patient by patient type derived from multiplying the single accesses frequency by its time/cost., and annual MF management for the “average center” patient cohort. Additionally, results are also presented for the three clinical status categories (non-anemic, anemic without transfusion need, and anemic with transfusion need), obtained as a weighted average of the corresponding patient types’ information, to better identify the impact of MF-related anemia and transfusions.

As a secondary outcome, the project also explored the patient perspective, specifically regarding their perception of the impact of MF-related anemia and transfusions on their social, work and daily lives, and satisfaction with hospital experience.

#### 2.4.1 Patient pathway times

Patient times included the active and waiting times as well as travel time for each access type. Active time is considered when undergoing an activity to actively manage the patient (e.g., during the visit). In contrast, waiting time refers to the time spent between activities with no added value to the process. Average travel time was derived from two sources: patient questionnaires, which provided transportation times from the patient’s residence to the hospital, and assumptions, such as estimating a 15-minute travel time from the patient’s residence to a nearby center for routine (non-transfusion-related) blood tests.

#### 2.4.2 Personnel time

The healthcare personnel time for each access type of the MF patient pathway was calculated based on the data provided by clinicians for each activity for each access and assigned to the main figures involved (i.e., hematologists, transfusion specialists, nurses, and administrative staff).

#### 2.4.3 Direct costs for the hospital and the NHS

Direct hospital costs for each access type included, when applicable, healthcare personnel costs, blood bag costs, and overhead costs. The personnel cost for each access was calculated using the average time absorbed during the different activities by each professional involved and their average hourly full hospital costs. The latter was calculated multiplying first the annual gross salaries (taken from the literature: Hematologist/Transfusion specialist: €75,000 (28), Nurse: €29,233 (29), and Administrative staff: €24,300 (30)) by a 1.4 multiplier (+40%) to account for indirect expenses (data provided by the payroll experts of Business Integration Partner S.p.A), including social security and insurance costs, severance pay and eventual benefits. Then the average hourly cost was calculated by dividing the average annual full hospital cost for 220 working days of 8 h. For the blood bag cost we considered the national tariff priced at €188.50 per unit (31). As regards overhead costs, a literature reference of €65.43/hour (32) was used for the use of one ‘chair hour’, including costs relating to common, general, management and administrative expenses, depreciation, non-health goods and non-health services. Overhead costs was only considered for accesses with transfusion, which we considered to be an accurate approximation, given that the chair time for transfusions has the greatest impact on the patient pathway.

#### 2.4.4 Indirect costs for patients and caregivers

Social costs related to lost productivity for both patients and caregivers were calculated using the average gross annual income in Italy of €30,838 (33). The average hourly cost of 17.52€ for patients and caregivers, obtained by dividing the gross annual average salary for 220 working days of 8 h, was applied to the total time spent (active, waiting and travel times) on each type of access. Productivity losses were first assessed for a single working patient and caregiver, then extrapolated the results to the average center cohort. To ensure greater accuracy, the analysis considered only the employed proportion of patients (36%) and accompanying caregivers (63%) within the average cohort. Additionally, the estimate of caregivers’ productivity losses was refined by factoring in the proportion of patients who are accompanied by at least one caregiver for their accesses (47%), and the average number of accompanying caregivers per patient (1.2).

All these parameters were determined by averaging the data reported by the clinicians interviewed or by the patients’ questionnaire (as detailed in **Supplementary Table 1**).

#### 2.4.5 Patient perspective

The questionnaire to patients (see **Supplementary Material B**) was designed to capture patients’ insights into the personal and social challenges of managing MF and its complications. Only a subset of questions was included in this analysis, while the complete questionnaire was used for other purposes, such as generating a call to action. For this analysis, patients were asked:

1. Average impact of anemia on working, social and daily life: “How much does anemia interfere with your working/social/daily life?” (Responses ranged from 1 (Not at all) to 5 (A lot)). The results were averaged according to patients’ self-reported DIPSS risk categories (low, medium, high) to capture differences in perceived impact across risk groups.

Additional questions were directed specifically to patients who indicated they had needed at least one transfusion in the past year:

2. Average impact of transfusions on working, social and daily life: “How much do transfusions interfere with your working/social/daily life?” (Responses ranged from 1 (Not at all) to 5 (A lot)).

3. Perceived level of adequacy during the day in hospital to receive transfusions: “In relation to your days in hospital to perform the transfusion, do you consider the following aspects adequate (waiting times; total time spent in the hospital; perceived level of organization; support of the healthcare personnel)?” (Responses ranged from 1 (Inadequate) to 5 (Very adequate)).

4. Evaluation of the overall hospital experience by transfused patients: “How do you overall rate your experience in the hospital to perform the transfusion?” (Responses ranged from 1 (Very poor) to 5 (Excellent)).

For these questions, results were averaged separately based on patients' transfusion dependency, to provide insights into any differences in their experiences.

## 3 Results

The results section is structured as follows: (i) patient questionnaire sample size, (ii) the average center and patient types’ pathway, (iii) patient pathway times, (iv) personnel time, (v) direct costs for the hospital, (vi) social impact, and (vii) patient perspective.

### 3.1 Patient questionnaire sample size

Responses were received from 191 Italian patients with a confirmed MF diagnosis. The geographic distribution of responses covered almost every Italian region, except for Trentino Alto Adige and Valle D’Aosta, grouped into four macroareas: North (32%), Center (25%), South (28%), and Islands (15%), reflecting effective outreach by AIPAMM. Most respondents (77%) were aged 18-65, while 22% were over 65, representing a younger-than-typical MF sample, likely due to the online format. Employment data showed 52% of respondents were employed, 26% retired, 7% unemployed, and 15% in other non-specified statuses.

Regarding DIPSS risk level, 35% reported to be low-risk, 42% intermediate-risk, and 13% high-risk with 10% unable or unwilling to report their risk level. This self-classification may reflect patients’ own awareness of their MF risk status. **Table 2** summarizes the relation between self-reported DIPSS risk, anemia prevalence, and transfusion needs. From the 170 patients who answered the two status-related questions, 56% reported anemia, and 16% had received at least one transfusion in the past year.

**Table 2:** Stratification of the average center MF patients based on their clinician reported DIPSS Risk and their clinical status and number of annual accesses for each of the 9 patient types.

DIPSS risk (%; NP)	Clinical Status (%; NP)	Patient Type	Access type and frequency			
			MF check-up	Combined check-up and transfusion	Transfusion-only	Blood test only
Low (28%; 29.4)	No anemic (93%; 27.4)	1	3.8			2.7
	Anemic, without need for transfusion (6%; 1.7)	2	3.8			2.7
	Anemic, need for transfusion (1%; 0.3)	3	3.6	0.2	0.2	2.3
Intermediate I (43%; 45.4)	No anemic (70%; 32.0)	4	6.4			
	Anemic, without need for transfusion (16%; 7.2)	5	6.4			
	Anemic, need for transfusion (14%; 6.3)	6	3.6	2.8	2.8	
Intermediate II / High (29%; 30.1)	No anemic (27%; 8.3)	7	12.9			
	Anemic, without need for transfusion (30%: 9.0)	8	12.9			
	Anemic, need for transfusion (43%; 12.8)	9	6.5	6.5	14.8	

MF Myelofibrosis DIPSS Dynamic International Prognostic Scoring System NP Number of Patients.

### 3.2 The average center and patient types’ pathways

The average center manages 105 MF patients, stratified by clinician-reported DIPSS risk (low, intermediate I, intermediate II/high) and clinical status (non-anemic, anemic without need for transfusions and transfusion-need), as described in Section 2.2 of the Methods. **Table 1** outlines the stratification of the MF cohort based on clinician reported DIPSS risk and clinical status, and the frequency and distribution of the four access types across the nine patient types in a year. For MF check-ups, 90% of patients follow a two-day pathway with blood tests done at local labs, while the other 10% do it at the hospital on the day of the visit. For transfusions, 68% complete the process in one day, and 32% follow a two-day pathway with pre-transfusion tests at the same facility. Regarding patient demographics, 35% are aged between 18 and 65 years, while 65% are over 65. Additionally, 37% of patients are employed, and 47% are accompanied by at least one caregiver during their visits. Among those accompanied, an average of 1.2 caregivers per patient is reported per access, and 63% of these caregivers are employed.

These data were obtained from clinicians based on their overall MF patient cohort, without stratification by DIPSS risk level or anemia-related clinical status. As a result, variations in age and employment distributions within specific subgroups (“Patient types”) could not be analyzed. No additional patient characteristics (e.g. gender, ethnicity) were collected.

### 3.3 Patient pathway times

**Figure 1:**
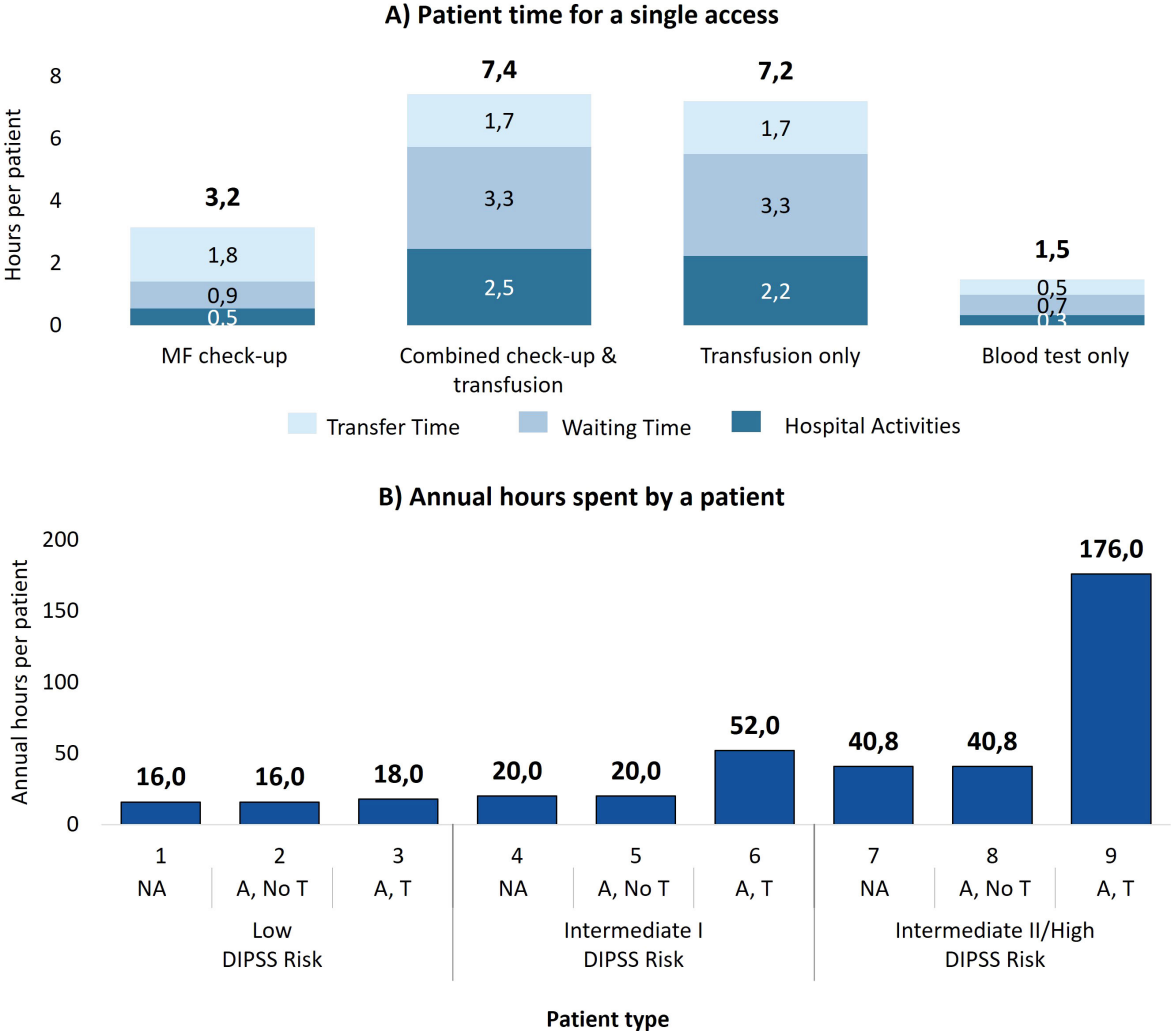
**(A)** Time dedicated by the patient for each type of access, divided into activity time, waiting time, and transfer time. **(B)** Time spent by a patient with myelofibrosis in one year of care for each patient type. NA, No anemic; A, No T, Anemic, without need for transfusion; A, T, Anemic, need for transfusion.

**Figure 1A** shows the average times spent by patients for the different access types to manage MF. Blood tests only are the least time-consuming, averaging 1.5 hours per visit, followed by MF check-up visits with 3.2 hours. Transfusion-related accesses are the most time-intensive (7.4 hours for combined check-up and transfusion accesses, and 7.2 hours for transfusion-only accesses), highlighting the high management burden for these patients. In addition, waiting time accounts for 44% of the total time for these accesses, suggesting potential inefficiencies in the process.

By multiplying the time per access by the number of annual accesses necessary for the 9 patient types, **Figure 1B** presents the annual time spent on MF management per patient type (e.g. on average patient type 6 follows 3.6 MF-check up accesses (3.2 hours per access), 2.8 combined check-up and transfusion accesses (7.4 hours per access) and 2.8 transfusion-only accesses (7.2 hours per access), which results in 52 annual hours spent in total). It is evident that the time commitments increase with disease severity, especially with the onset of transfusion-dependence. Low-risk patients spend a minimum of 16 hours per year and up to 18 hours in the rare cases in which they require transfusions. The management burden worsens in patients at intermediate risk I (min. 20 hours; max. 52 hours) and increases drastically for patients at intermediate II/high risk: 40.8 hours for those who do not receive transfusions and 175.5 hours for transfusion dependent patients.

Aggregating the data by clinical status (**Table 3**) reveals that a patient who needs transfusions spends, on average, more than six times as much time managing myelofibrosis as a patient without anemia, and four times as long as an anemic patient without transfusions.

**Table 3:** Economic-organizational results aggregated by clinical status for a single patient and for all patients in the average center.

		Clinical status group		
		No anemic	Anemic, without need for transfusion	Anemic, need for transfusion
**For a single patient**	Patient times [hours/year]	21	30	133
	Personnel time [hours/year]	4	5	17
	Hospital/HNS direct cost [€/year]	165	249	6,603
	Social Costs [€/year]	367	528	2,332
**For all patients in the average center**	Patient times [hours/year]	1,417	537	2,590
	Personnel time [hours/year]	237	92	331
	Hospital/HNS direct cost [€/year]	11,173	4,433	128,504
	Social Costs [€/year] – Working patients and caregivers	17,532	6,859	33,293

Considering the 105 patients with MF managed annually in the average center, it is estimated that for a year of treatment, patients collectively invest a total of 4,543 hours for the management of MF. The distribution among the three clinical status groups is summarized in **Table 3**, showing that, despite representing only 18% of the patients, anemic patients requiring transfusion cover 57% of the total hours spent annually by all patients managed.

### 3.4 Personnel time

**Figure 2:**
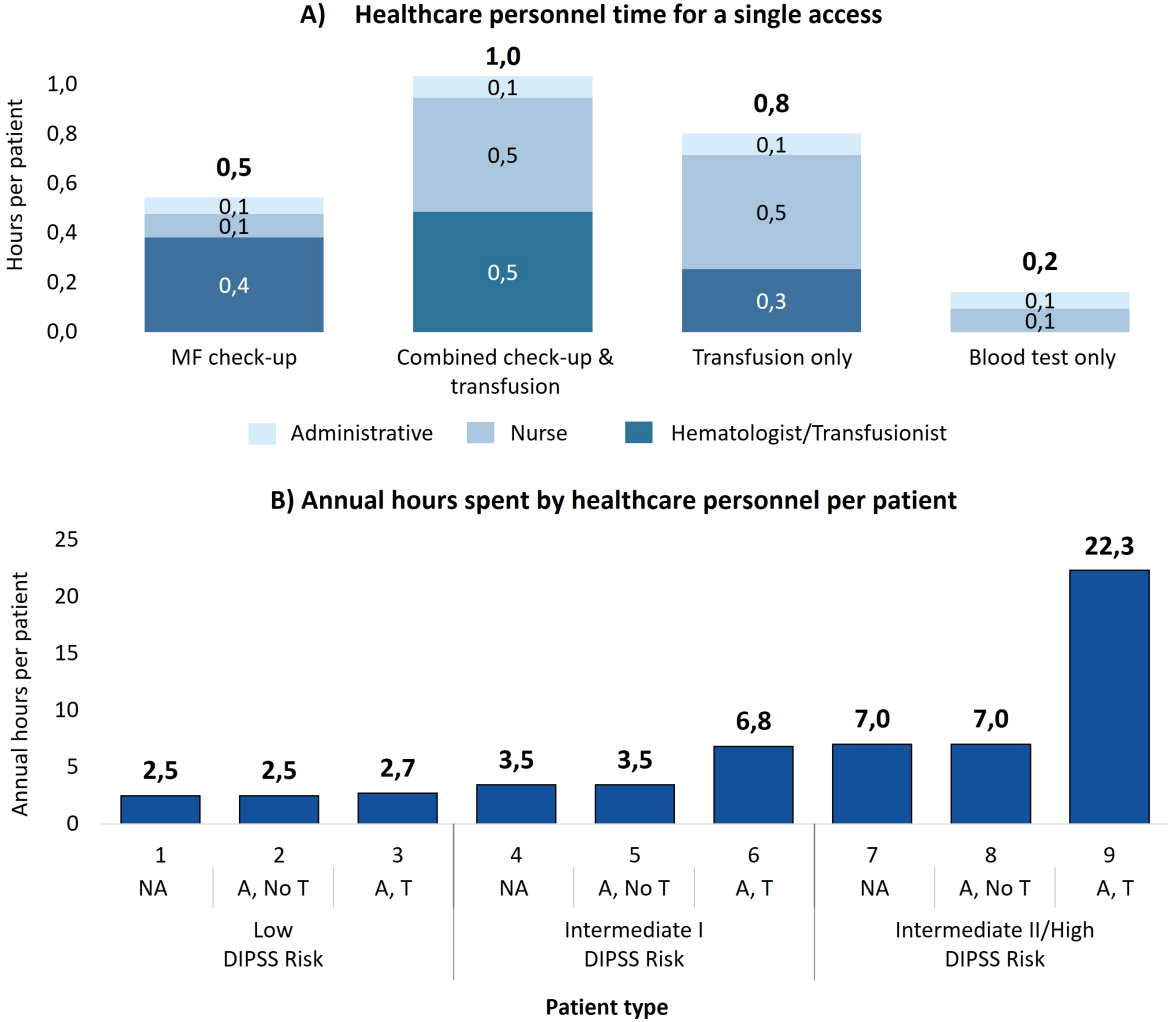
**(A)** Healthcare personnel time to manage each single access, divided by the contribution of each professional figure involved. **(B)** Annual time spent by healthcare personnel to manage a patient with myelofibrosis for each patient type. NA, No anemic; A, No T, Anemic, without need for transfusion; A, T, Anemic, need for transfusion.

On average, MF check-up visits require 33 minutes of active personnel time, while combined check-up and transfusion accesses demand 62 minutes. For transfusion-only accesses, the time reduces to 48 minutes due to the absence of MF treatment monitoring activities, focusing solely on transfusion eligibility. Blood tests only require just 10 minutes of personnel engagement per visit. **Figure 2A** illustrates the distribution of time across various personnel for each access type.

Over the course of a year, the cumulative personnel time per patient varies significantly depending on the patient type and as illustrated in **Figure 2B**, the time dedicated to patient management increases as the DIPSS prognostic risk increases and as the patient’s clinical status worsens, from the patient without anemia to the patient with anemia and the need for transfusions. For patients at intermediate II/high risk, personnel spend 7.0 hours for those who do not receive transfusions and 22.3 hours for transfusion dependent patients. As summarized in **Table 3**, when aggregated by clinical status, non-anemic patients require 3.5 hours annually, anemic patients without transfusions require 5.2 hours, and transfusion dependent patients require 16.9 hours. Therefore, a patient requiring transfusions requires almost five times as much hospital staff time annually to manage as a non-anemic patient. Compared to a patient with only anemia, the time required is more than three times as long.

Considering all the 105 patients managed annually at the average center, total personnel time reaches 660 hours per year. **Table 3** details the breakdown: non-anemic patients, who constitute 65% of the cohort, account for 237 hours (36% of the total time), while transfusion dependent patients, representing only 18% of the cohort, require 331 hours (50% of the total time). This highlights the disproportionate resource burden caused by the care of transfusion dependent patients.

### 3.5 Direct costs for the hospital and the NHS

**Figure 3:**
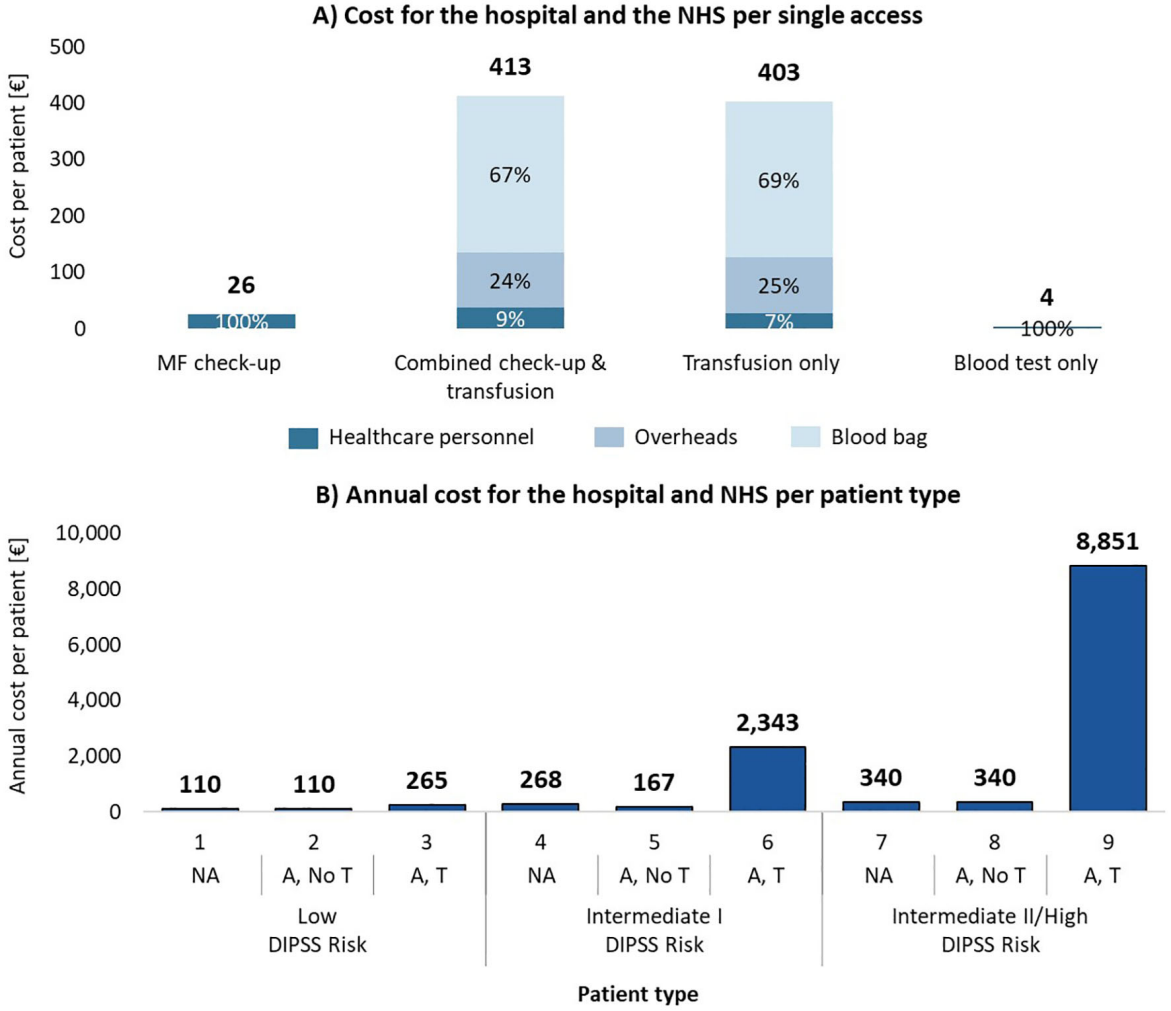
**(A)** Hospital and NHS cost per patient for each type of access, consisting of hospital personnel costs, blood bag costs and overhead costs (only for transfusions). **(B)** Total annual cost for one year of care per patient type. NA, No anemic; A, No T, Anemic, without need for transfusion; A, T, Anemic, need for transfusion.

Transfusion-related accesses represent a substantial financial burden in comparison to the other ones, as shown in **Figure 3A**. Combined check-up and transfusion accesses incur an estimated care cost of €413 per access, while transfusion-only accesses cost €403. In contrast, care costs are significantly lower for blood tests only (€4) and MF check-up visits (€26). The predominant contributor to the higher costs in transfusion-related accesses is the blood bag, which accounts for 67%-69% of the total estimated care cost per transfusion. Additionally, structural overheads, at €99 per transfusion access, make up 24%-25% of the overall cost.

**Figure 3B** illustrates the annual estimated care costs per patient type, showing the sharp increase associated with transfusion dependency. Low-risk non-anemic patients have an estimated care annual cost of €110, while transfusion dependent patients at intermediate II/high risk see care costs escalate to €8,850. When aggregated by clinical status (**Table 3**), the estimated annual care cost for a non-anemic patient is €165, for an anemic patient without transfusions is €249, and for a transfusion dependent patient is €6,603. This reflects a significant financial burden associated with transfusion dependency, which is over 40 times higher than the care cost for non-anemic patients.

In the hypothetical average center, managing 105 MF patients annually results in total estimated hospital and NHS care costs of €144,064 per year. As shown in **Table 3**, costs are heavily skewed toward the care of transfusion dependent patients, who, despite representing only 18% of the patient cohort, account for 89% of the total costs (€128,458). In contrast, the estimated care costs for non-anemic patients (65% of the cohort) and anemic patients (17% of the cohort) without transfusions are €11,173 and €4,433, respectively. This demonstrates the significant economic impact of managing transfusion dependent MF patients.

### 3.6 Social impact (indirect costs) for patients and caregivers

**Figure 4:**
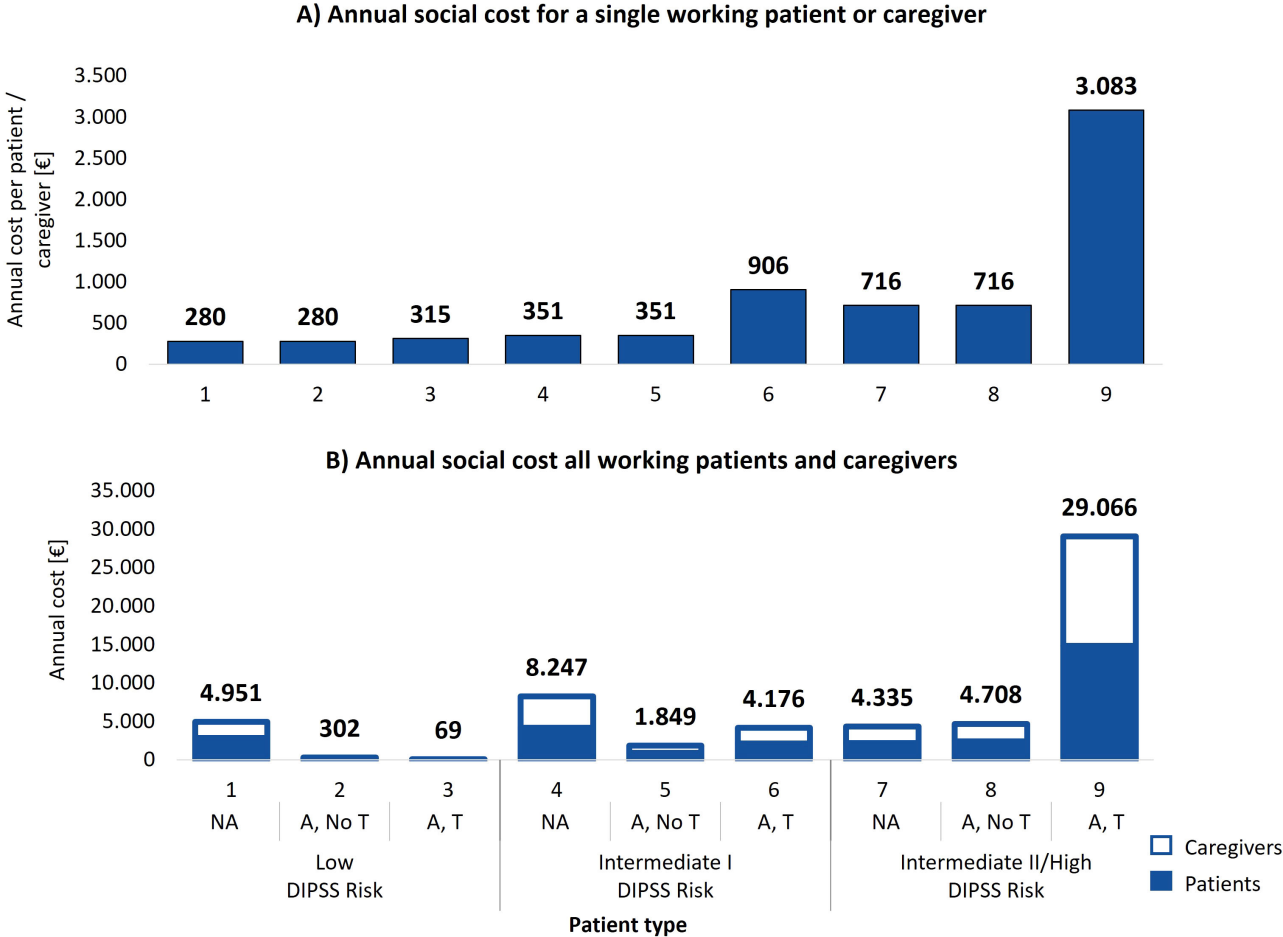
**(A)** Total annual estimated social cost incurred by a single working patient or caregiver per patient type. **(B)** Annual social costs experienced by working patients and caregivers from the average center distributed across the 9 patient types. NA: No anemic; A, No T: Anemic, without need for transfusion; A, T: Anemic, need for transfusion.

The economic impact of managing MF extends beyond healthcare facilities, involving significant productivity losses for patients and caregivers. A low-risk non-anemic working patient or caregiver experiences the lowest productivity loss, with estimated indirect costs ranging from €280 to €315 annually, depending on transfusion needs as shown in **Figure 4A**. In contrast, intermediate II/high-risk transfusion dependent patients face social care costs rising up to €3,083 per year.

When analyzing social care costs by clinical status (**Table 3**), a non-anemic working patient or caregiver incurs an average estimated annual indirect care cost of €367, an anemic patient without transfusions incurs €528, and a transfusion dependent patient faces €2,332. This demonstrates the significant escalation of social costs with the onset of transfusion dependency, with transfusion dependent patients facing indirect care costs over six times higher than non-anemic patients.

Overall, considering all working patients and caregivers of the average center, the total estimated social care cost amounts to €57,684. **Figure 4B** illustrates the annual social costs for all working patients and caregivers by patient type group. Low-risk non-anemic patients account for €4,951 annually, while transfusion dependent patients at intermediate II/high risk incur care costs rising to €28,989. As shown in **Table 3**, transfusion dependent patients and their caregivers represent 58% of the total estimated social care costs (€33,293), despite representing only 18% of the patient cohort. These findings highlight the disproportionate social cost burden associated with the care of transfusion dependent patients and their caregivers.

### 3.7 Patient perspective

**Figure 5:**
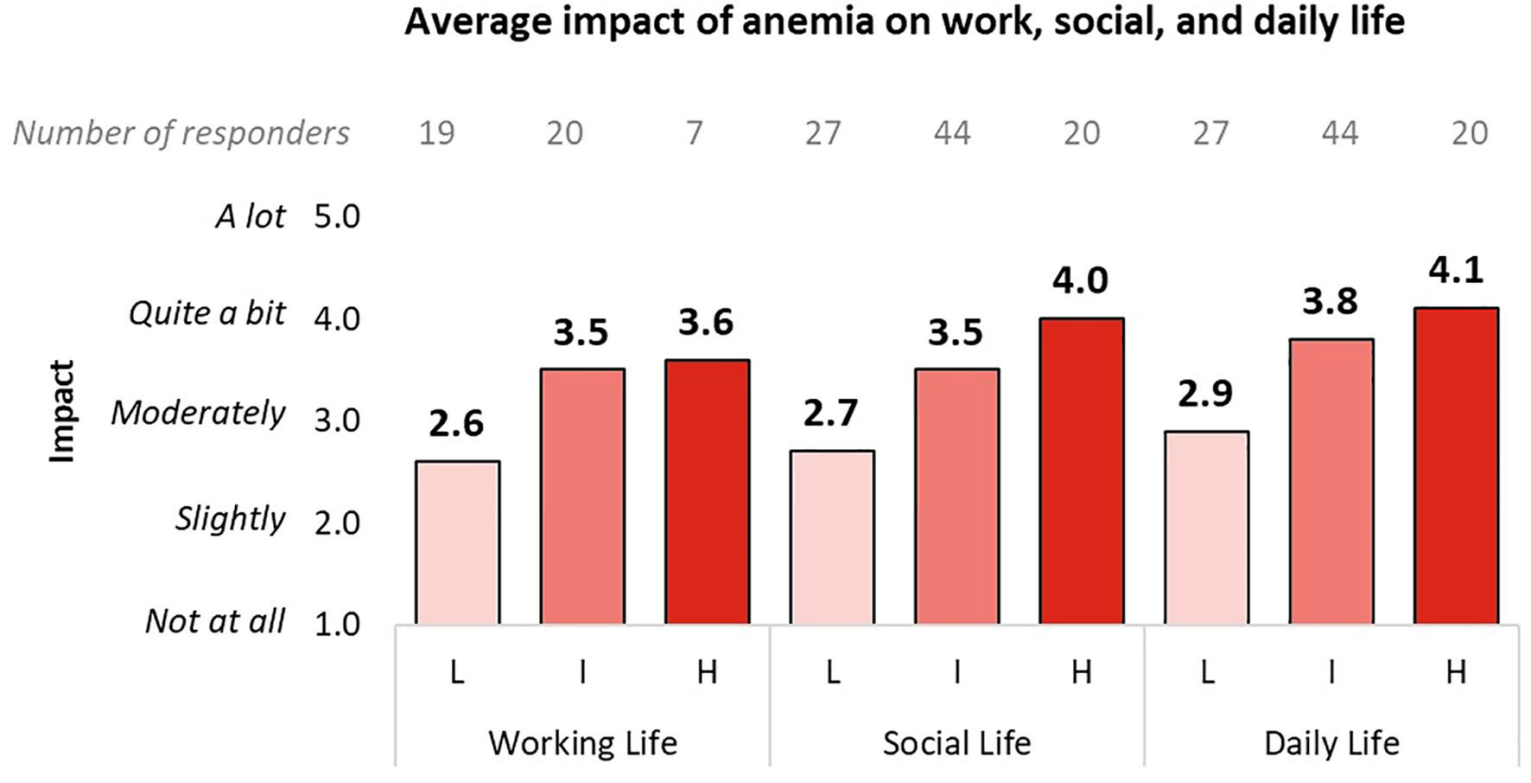
Impact of anemia on the work, social, and daily life of patients, divided into subgroups according to the DIPSS classification. Only responses from working patients were analyzed for the work life question. L, Low DIPSS risk; I, Intermediate DIPSS risk; H, High DIPSS risk.

**Figure 5** presents the patient-reported impact of anemia on working, social, and daily life across different DIPSS risk categories, highlighting the increasing burden of anemia as disease severity progresses. Low-risk patients reported moderate interference, with scores ranging from 2.6 to 2.9 across all domains. Intermediate-risk and high-risk patients reported higher interference, particularly in daily life, where high-risk patients scored 4.1 out of 5.

**Figure 6:**
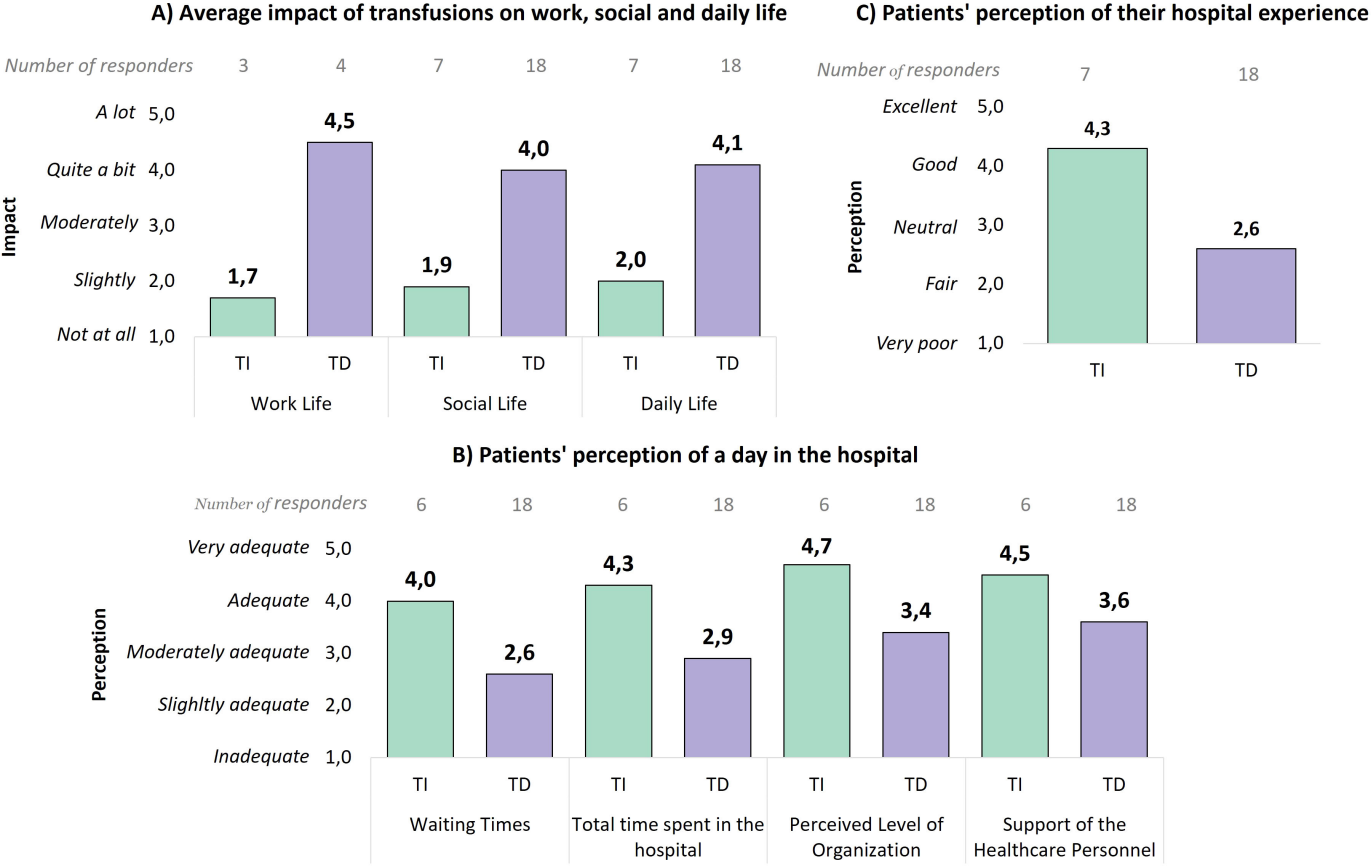
**(A)** Average impact of transfusions on the work, social, and daily life of patients, divided between non-transfusion dependent and transfusion dependent patients. Only responses from working patients were analyzed for the work life question. **(B)** Perceived adequacy level during the hospital day for receiving transfusions, divided between non-transfusion dependent and transfusion dependent patients. **(C)** Evaluation of the hospital experience by patients, divided between non-transfusion dependent and transfusion dependent patients. TI, Transfusion-independent; TD, Transfusion dependent.

For patients who required at least one transfusion in the past year, **Figure 6A** highlights the effect of transfusions on life domains. Non-transfusion dependent patients reported minimal disruption (working life: 1.7, social life: 1.9, daily life: 2.0), whereas transfusion dependent patients experienced significant interference, with scores of 4.5 for working life and over 4 for both social and daily life. These results emphasize the considerable lifestyle impact associated with transfusion dependency.

The patient experience also varied considerably between transfusion dependent and non-dependent patients. Non-transfusion dependent patients rated their experiences more positively (**Figure 6B**), with high scores for waiting times (4.0), total length of stay (4.3), organizational level (4.7), and support from healthcare personnel (4.5). On the contrary, transfusion dependent patients provided lower ratings, particularly for waiting times (2.6) and total length of stay (2.9). Moreover, the average overall hospital experience rating (**Figure 6C**) was particularly lower for transfusion dependent patients (2.6) compared to non-transfusion dependent patients (4.3), emphasizing the significant disparity in patient satisfaction.

Overall, these findings show the high burden of managing MF, especially for patients requiring regular transfusions, and highlight areas that could be improved to enhance their overall experience.

## 4 Discussion

The BEAT project aimed to examine the economic and organizational impacts of myelofibrosis on patients, caregivers, and healthcare providers in Italy, with a specific focus on anemia and transfusion dependency.

The results of this project demonstrate that transfusion dependency in patients with MF significantly increases disease burden, time spent by patients and healthcare personnel for disease management, and economic costs for both patients and the Italian NHS.

Patients undergoing MF management dedicate a substantial amount of time to their care, which increases with disease severity. Transfusion dependent patients spend up to 133 hours per year on disease management, four times the time required by anemic patients without transfusions and six times that of non-anemic patients. Waiting time accounts for almost half (44%) of the total time required for transfusion accesses. Despite some waiting being inevitable, this result suggests potential inefficiencies in the process. These inefficiencies could be at least partially addressed through a Lean approach, which focuses on eliminating waste and improving process efficiency (34). Key strategies could include implementing punctual scheduling of patient arrivals based on the expected duration of their treatment, and splitting care over two days, with blood tests and transfusion preparation conducted the day before transfusion (e.g. blood crossmatching). These measures would help reduce non-physiological patient waiting times, enhancing not only patient experience but also allowing hospitals to optimize resource allocation (34).

As expected, managing patients with severe MF entails higher organizational and economic burdens. As highlighted in **Table 1**, patients with intermediate II/high risk MF require a considerably greater number of check-ups, even when transfusions are not necessary (12.9 checkups/year for intermediate II/high risk compared to 3.8 and 6.4 check-ups/year for low- and intermediate I- risk patients, respectively). Consequently, patients in higher-risk groups spend more time managing their care compared to those in lower-risk groups, regardless of their anemia-related clinical status. Similarly, when comparing intermediate II/high-risk MF patients without anemia to low-risk MF patients with anemia who require transfusions, the latter still have fewer total accesses, even when accounting for blood test-only visits and transfusion-related appointments. This is because low-risk anemic patients undergoing transfusions typically require only an average of 0.4 transfusions per year (see **Table 1**) and are not transfusion-dependent, unlike patients in higher-risk categories. Additionally, the overall times for blood test-only accesses (1.5 hours of patient time and 0.2 hours of personnel time per access) is lower than for check-ups (3.2 hours and 0.5 hours, respectively, **Figures 1**, **2**).

Nevertheless, our analysis also reveals that transfusion dependency requires over three times more personnel time (up to 22.3 hours/patient/year) than patients with similar risk levels who do not require transfusions (**Figure 2B**). This translates to significantly higher direct care costs for the NHS as well as social costs (productivity loss) for transfusion dependent patients (especially those in the intermediate I and intermediate II-high risk groups) and their caregivers.

It is important to note that the true costs of managing patients in higher risk categories, particularly those with transfusion dependency, are likely even greater than those reported in this study, as factors such as managing complications and the costs of pharmacological treatments and medications were not included in our analysis.

Our findings are in line with other previously published studies, which consistently indicate higher care costs as the severity of anemia increases (27, 35–37). However, study settings and estimated burdens vary between studies (38–41).

A recently published retrospective study also shows increased healthcare (medical and pharmacy) costs associated with worsening anemia and transfusion dependency (35). However, this study is based on U.S data and focuses exclusively on costs. In contrast, our analysis incorporates the broader burden of the disease, not only in terms of direct costs but also evaluating the time required for disease management. Additionally, our project is based on data from Italian centers, which can differ substantially from the U.S. model, providing unique insights into the burden of MF within a European healthcare context.

Previously, another Italian study attempted to quantify the financial impact of MF on patients and caregivers (27). This study used a questionnaire to estimate direct healthcare access costs (including transportation, examinations, and tests) as well as costs associated with informal caregiving and income loss.

To the best of our knowledge, however, our project is the first to quantify the time and costs associated with MF management, and particularly with RBC transfusion, for both MF patients/caregivers and the Italian NHS.

The results from our model analysis, based on data collected from the clinicians’ interviews, were further validated, and enriched by the data gathered via the patient survey. The geographic distribution of responses covered nearly all Italian regions, with only two underrepresented regions making up just 2.01% of the population (42), which allowed for a robust representativeness of the MF patient population across Italy. In fact, our survey confirmed that, from the patient perspective, anemia and transfusion dependency substantially increase the burden of illness, affecting daily activities as well as social and work life (**Figures 5**, **6**). Beyond the raw time and cost figures, these findings allow us to better contextualize the broader impacts of transfusion dependency illustrating the lifestyle burdens. Additionally, our survey reveals that the impact of anemia increases as the disease progresses (from low to high risk, **Figure 5**). However, this result could be at least partially explained by the growing proportion of transfusion dependent patients as the disease advances. Finally, compared to transfusion-independent patients, those who are transfusion dependent report a worse perception and evaluation of their hospital experiences, likely due to the frequency and duration of hospital accesses required to manage their condition. In particular, transfusion dependent patients express higher dissatisfaction concerning waiting times and overall organization, which is in line with the high weight (44%) of waiting time on the overall transfusion access time result discussed before.

The results of this project should be considered in light of some limitations. First, all data were self-reported by respondents without cross-verification through real-world evidence, which could introduce reporting bias. Additionally, the time spent by healthcare professionals other than hematologists was assumed based on hematologists’ estimates, potentially overlooking variations in other roles. However, the data provided by the clinicians were consistent and aligned across them, ensuring a reliable foundation for the analysis. The clinicians and centers involved were carefully selected to be representative of the Italian MF treatment landscape. While the generalizability of their responses to all Italian centers may be somewhat limited, the chosen centers provide a reasonable cross-section of MF practices across Italy. Similarly, while the patient questionnaire data could not be directly cross-validated with clinician data due to the lack of tracking of treatment centers, it still offers a broad and relevant perspective on the patient experience. Additionally, the project focused on the organizational and economic costs for the NHS associated with MF and MF-related anemia, excluding treatment costs for both MF and MF-related anemia. Since the primary objective was to examine the social and economic burden of transfusion-based anemia management, the number of patients undergoing pharmacological treatments for anemia, either alone or in combination with transfusions, was not investigated. Consequently, the costs associated with these pharmacological treatments, and how they compare to the costs of transfusion-based management, could not be assessed within the scope of this study. Lastly, the analysis of social costs is limited to indirect costs related to productivity loss for patients and caregivers, without accounting for other indirect costs, such as travel expenses, out-of-pocket payments, and the potential need for paid professional caregivers.

To address these limitations, further studies should aim to validate self-reported data by cross-referencing with hospital records and employing methodologies like time-motion studies for more precise measurement of patient and personnel times. A broader range of centers should be involved to improve the generalizability of the results across Italy, and it would be beneficial to interview other healthcare personnel. Distributing patient questionnaires directly within the interviewed centers would not only yield a more representative sample of the overall MF patient population, overcoming the potential bias toward younger patients observed with the online format used in this project, but also enhance the robustness of the findings by better aligning the patient and clinician data. Moreover, a more comprehensive range of social costs and treatment costs should be incorporated to provide a fuller understanding of the economic impact on patients and families and the NHS. However, this project serves as the first building block in understanding the economic and organizational burden of MF-related anemia and transfusions. By focusing on a conservative approach with a minimum set of costs and time-related factors, the results presented likely underestimate the true impact. Future studies building on these findings will provide a more comprehensive picture of the multifaceted burden faced by MF patients and the NHS, thus further highlighting the value of this initial work.

## 5 Conclusions

Our findings show that as patients’ clinical status worsens, the burden on both the patients and the NHS becomes progressively more pronounced. Transfusion dependent patients spend up to 176 hours annually on MF care, which is more than six times the time spent by non-anemic patients, and four times that of anemic patients without transfusions. Additionally, transfusion dependency carries a financial burden over 40 times higher than that for non-anemic patients.

These findings therefore confirm and emphasize the substantial and multifaceted burden of myelofibrosis-related anemia, with transfusion dependency representing a significant challenge for patients, caregivers, and healthcare systems. This burden underscores an urgent need for targeted strategies to improve the management of MF, mainly anemia and transfusion needs. Innovations should focus on both therapeutic advances, such as disease-modifying and novel anemia-targeting therapies, and systemic improvements, including optimizing hospital workflows to enhance efficiency. While the duration of transfusion administration is biologically fixed, addressing avoidable inefficiencies in processes such as scheduling and preparation could minimize patient waiting times without disrupting clinician utilization. Reducing non-value-adding wait times could improve the overall patient experience and lessen the logistical burden on them.

Together, these measures could help mitigate the economic strain on healthcare systems and lessen the time-related and emotional impact on patients and caregivers, ultimately enhancing quality of care and patient outcomes.

## Data Availability

The raw data supporting the conclusions of this article will be made material available upon request to interested researchers.
