# Supplementary material for: Anemia and blood transfusions in myelofibrosis: economic and organizational impact on Italian patients, caregivers and hospitals
Source: Front Oncol. 2025 Mar 7;15:1549023. doi: 10.3389/fonc.2025.1549023 (PMC11926708; doi:10.3389/fonc.2025.1549023)
Supplement: Supplementary file 1 [file DataSheet1.zip › Data Sheet 1/Supplementary Figure 1.pdf]

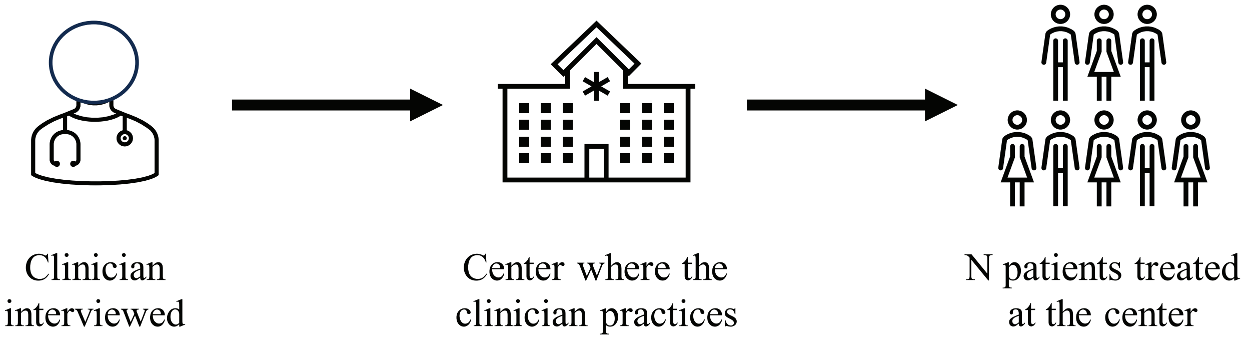

$$\bar{X} = \frac{(x_1 \cdot n_1) + (x_2 \cdot n_2) + \cdots + (x_{14} \cdot n_{14})}{(n, +n_2 + \cdots + n_{14})}$$

where:

$\bar{X}$ is the average value used for the average center;
$\mathbf{x_1}$ is the value gathered by clinician 1;
$\mathbf{n_1}$ is the number of patients treated at clinician 1's center.

Example:

Clinician 1's center treats 100 patients, of which 32% are at low risk. Clinician 2's center treats 50 patients, of which 29% at low risk.

The % of patients at low risk treated in the average center would be = (100*32%)+(50*29)/(100+50) = 31%.

Supplementary Figure 1. Schematic representation of the method used to calculate weighted average values. Data on the number of patients managed by each of the 14 clinicians' centers were collected (illustrated in the schematic at the top). These patient numbers were used to compute weighted average values, following the formula provided. At the bottom, an example demonstrates how a weighted average value is derived using this approach. A comprehensive list of parameters used to define the 'average center,' along with the calculation methods, is detailed in Supplementary Table 1.
