# Supplementary material for: Anemia and blood transfusions in myelofibrosis: economic and organizational impact on Italian patients, caregivers and hospitals
Source: Front Oncol. 2025 Mar 7;15:1549023. doi: 10.3389/fonc.2025.1549023 (PMC11926708; doi:10.3389/fonc.2025.1549023)
Supplement: Supplementary file 1 [file DataSheet1.zip › Data Sheet 1/Supplementary Material A.pdf]

Supplementary Material A | Clinician Interview

1. CENTRE

IDENTITY CARD

1.1 TRANSFUSION PLACE

Indicate where transfusions are carried out

Place

1.2 HAEMATOLOGICAL CHECK-UP

Indicate where hematological check-ups are carried out

Place

1. ACCESSES

VOLUMES

2.1 VOLUMES

Indicate the **number of MF patients** treated in the last 12 months:

MF Patients

2.2 DIAGNOSTIC CLASSIFICATION (DIPSS)

Out of 100 MF patients, please indicate the percentage of patients classified as low, intermediate, and high risk.*

Low		Intermediate I		Intermediate II and High	
	%		%		%

2.3 ANEMIA AND TRANSFUSION DEPENDENCE

Out of 100 MF patients, please indicate the percentage affected by anemia and transfusion dependency based on risk classification

	**Low**		**Intermediate I**		**Intermediate II and High**	
With anemia		%		%		%
Transfusion addicts		%		%		%

*Assessment of the level of severity of the pathology based on clinical-biological variables using the Dynamic International Prognostic Scoring System

2.4 AGE OF MF PATIENTS

Out of 100 MF patients referred to the onco-hematology center, what is the **percentage for each age group**?

< 18 years old		%
18 – 65 years old		%
> 65 years old		%

2.5 MF PATIENTS ACCESS

Indicate the **average total number of check-up and transfusions per year** for MF patients based on their risk classification

	**Low**	**Intermediate I**	**Intermediate II and High**
Number of MF check-ups			
Number of trasfusions			

2.6 WORKING PATIENTS

What is the percentage of **working patients**?

Number of **working patients** ____ %

2.7 ACCOMPANIED PATIENTS

What is the percentage of **accompanied patients?**:

	**Low**	**Intermediate I**	**Intermediate II and High**
For transfusions			
For check-ups			

2.8 WORKING CAREGIVERS

What is the percentage of **caregivers of working age (from 16 to 65 years)?**

Working caregivers ____ %

3.1 NUMBER OF BLOOD TESTS

Indicate the **average number of blood draws** performed annually for a MF patient:

	Non-Transfusion-Dependent	Transfusion-dependent
No. of samples		

3.2 PRE-TRNASFUTION CONTROL PLACE

Indicate the percentage of **MF patients** who follow **the pre-transfusion control in**:

Hospital		%
Own residence		%
Other place		%

3.3 PRE-TRANSFUSION CONTROL

Please indicate the percentage of **MF patients** who carry out the **pre-transfusion control during**:

The days **before** the transfusion		%
**On the day** of the transfusion		%

3.4 VOLUMES

Indicate the **average number of blood bags** prepared per patient for a transfusion sitting and the **average volume** of a bag:

Number of bags		
Average bag volume		ml

4.TIME

HOSPITAL PERSONNEL TIME

4.1 TIMES AND ACTIVITY ROLES:

Indicate the **average time for each activity**, if applicable, and the **primary professional figure responsible** for it

	Activity	Time		PROFESSIONAL FIGURE MAINLY INVOLVED IN THE ACTIVITY				WHERE IT TAKES PLACE
				Hematologist	Transfusionist	Nurse	Administrative	
Pre-Transfusion	Sampling		*Min*	☐	☐	☐	☐	
	MF-check-up		*Min*	☐	☐	☐		
	Pre-transfusion check-up		*Min*	☐	☐	☐		
Transfusion Day	Check-in		*Min*	☐	☐	☐	☐	
	MF-check-up		*Min*	☐	☐	☐		
	Blood bag preparation		*Min*	☐	☐	☐	☐	
	Blood bag delivery		*Min*	☐	☐	☐		
	Transfusion chair setting		*Min*	☐	☐	☐		
	Chair time for transfusion		*Min*	☐	☐	☐		
	Patient observation time		*Min*	☐	☐	☐		
	Discharge		*Min*	☐	☐	☐	☐	

4.3 ADMINISTRATION TIME

Indicate the **average duration** of the following **activities** according to the risk classification of MF patients, if applicable:

	Low	Intermediate I	Intermediate II and High	
Transfusion time				*Min*
Time at transfusion chair				*Min*
Average time spent in the medical center				*Min*

4.4 PATIENT WAITING TIME

Indicate the **average waiting times** for patients the different activities considering the perspective of MF patients

Waiting for acceptance		*Min*
Waiting for blood draw		*Min*
Waiting for blood draw results		*Min*
Waiting for MF check-up		*Min*
Waiting for pre-transfusion check-up		*Min*
Waiting for transfusion		*Min*
