# Supplementary material for: Anemia and blood transfusions in myelofibrosis: economic and organizational impact on Italian patients, caregivers and hospitals
Source: Front Oncol. 2025 Mar 7;15:1549023. doi: 10.3389/fonc.2025.1549023 (PMC11926708; doi:10.3389/fonc.2025.1549023)
Supplement: Supplementary file 1 [file DataSheet1.zip › Data Sheet 1/Supplementary Material B.pdf]

Supplementary Material B | Patient questionnaire

The questionnaire was transferred to an online platform for distribution. Automatic skip logic was implemented, meaning that based on responses to certain questions (e.g., question 1), only the relevant questions for each specific patient were automatically displayed.

BEAT Project: Survey for Patients with Myeloproliferative Diseases

1. Have you been diagnosed with any of the following myeloproliferative diseases?
   ◯ Yes, myelofibrosis ◯ Yes, myelofibrosis post-polycythemia vera ◯ Yes, myelofibrosis post-thrombocytemia
   ◯ No ◯ N/A

2. Indicate the age group you belong to
   ◯ Under 18 years ◯ Between 18 and 65 years ◯ Over 65 years ◯ N/A

3. Please indicate your region of residence
   Region: ______________________

4. Please indicate your employment status:
   ◯ Employee ◯ Currently unemployed ◯ Retired ◯ Other *(e.g. student, homemaker*
   ◯ N/A

5. In which risk classification of myelofibrosis do you fall?
   ◯ Low ◯ Medium ◯ High ◯ I don't know how to answer ◯ N/A

6. Have you been granted any of the following assistance benefits: disability pension, disability allowance, attendance allowance, attendance indemnity?
   ◯ Yes ◯ No ◯ N/A

7. Do you suffer from anemia (i.e. reduction in hemoglobin levels compared to normal values. It is indicated in the blood test report with an asterisk)?
   ◯ Yes ◯ No ◯ N/A

8. Was anemia diagnosed at the time of the disease diagnosis?
   ◯ Yes ◯ No ◯ N/A

9. If answered *«No»* to question 8: How long after the disease diagnosis was anemia diagnosed?
   ___________ months ◯ I don't know how to answer ◯ N/A

10. Based on your symptoms, how well do you believe the anemia is under control?
    ◯ Not at all ◯ Slightly ◯ Moderately
    ◯ Completely ◯ N/A

11. How much does anemia interfere with:

	Not at all	Slightly	Moderately	Quite a bit	A lot	N/A
Your work life	◯	◯	◯	◯	◯	◯
Your social/family life	◯	◯	◯	◯	◯	◯
Your daily life	◯	◯	◯	◯	◯	◯

Where written N/A, the actual option shown to the patient was: *I don't know/I don't want to answer*

BEAT Project: Survey for Patients with Myeloproliferative Diseases

12. Do you usually have blood tests and check-up on the same day?
    ◯ Yes ◯ No ◯ N/A

13. Do you usually have the blood tests at the same Hospital as the check-up or at a nearby blood collection point?
    ◯ Hospital ◯ Nearby blood collection point ◯ Other ◯ N/A

14. Could you estimate how many hours you spend on average in one month in healthcare facilities due to anemia from your arrival to your departure for blood tests and check-up?
    ________ hours ◯ I don't know how to answer

15. In the last 3 months, have you been hospitalized urgently due to anemia?
    ◯ Yes ◯ No ◯ N/A

16. If answered *«Yes»* to question 15: Could you estimate how many hours/days you spend on average in the hospital from your admission to your discharge for emergency hospitalizations?
    ________ hours
    ________ days
    ◯ I don't know how to answer

17. In the past year, have you received at least one blood transfusion?
    ◯ Yes ◯ No ◯ N/A

18 If you have received more than one transfusion, what has been the impact in terms of time spent (days)?
    ◯ Less than 5 ◯ Between 5 and 12 ◯ Between 13 and 23
    ◯ Between 24 and 36 ◯ More than 37 ◯ N/A

19. Are you currently receiving transfusions?
    ◯ Yes ◯ No ◯ N/A

20. Are you taking medication because of the transfusions?
    ◯ Yes, an iron chelator ◯ Yes, other ◯ No ◯ N/A

21. Do you go to the hospital alone or accompanied with someone for the transfusion?
    If accompanied, please indicate by how many people.
    ◯ Alone ◯ Accompanied by__________ people

22. Is your caregiver currently employed?

Caregiver 1	◯ Yes	◯ No	◯ N/A
Caregiver 2	◯ Yes	◯ No	◯ N/A

23. What mode of transportation do you usually use to go to the hospital?
    ◯ Taxi ◯ Car ◯ Bus / train
    ◯ Walking ◯ Other ◯ N/A

24. Usually, do you resume your work activities normally after the transfusion?
    ◯ Yes ◯ No ◯ I don't work ◯ N/A

25. If you answered *«No»* to question 24, how much time passes before returning to normal work routine?
    ________ hours
    ________ days
    ◯ N/A

Where written N/A, the actual option shown to the patient was: *I don't know/I don't want to answer*

BEAT Project: Survey for Patients with Myeloproliferative Diseases

26. How much do blood transfusions interfere with:

	Not at all	Slightly	Moderately	Quite a bit	A lot	N/A
Your work life	○	○	○	○	○	○
Your social/family life	○	○	○	○	○	○
Your daily life	○	○	○	○	○	○

27. In the last 3 months, how many days of work have you missed due to transfusions? ________ *days* ○ N/A

28. In the last 3 months, how many days of work have your caregiver(s) missed in total due to the transfusions? ________ *days* ○ N/A

29. When you are accompanied to the hospital, does your companion benefit from paid leave/permission from work (e.g., Law 104/92, leave for caregivers) ○ Yes ○ No ○ N/A

30. Could you estimate how many hours you spend on average in the hospital for the transfusion from your admission to your discharge? ________ hours ○ I don't know how to answer

31. In relation to your days at the hospital for transfusions, do you find adequate:

	Inadequate	Slightly adequate	Moderately adequate	Adequate	Very adequate	N/A
The waiting times	○	○	○	○	○	○
The duration of your stay	○	○	○	○	○	○
The level of organization (e.g., respect for the appointment time)	○	○	○	○	○	○
The support provided by the staff	○	○	○	○	○	○

32.

	Not at all	Slightly	Moderately	Quite a bit	Very much	N/A
• Are you satisfied with the organization and efficiency of your treatment pathway?	○	○	○	○	○	○
• Are you satisfied with the adherence to appointment times?	○	○	○	○	○	○
• Are you satisfied with the time dedicated to you by the doctor and nurses?	○	○	○	○	○	○
• Do you consider the length of stay in the hospital (from your admission to your discharge) adequate?	○	○	○	○	○	○

33. How would you rate your overall experience at the hospital for receiving transfusions?

○ Very Poor ○ Fair ○ Neutral ○ Good ○ Excellent

Where written N/A, the actual option shown to the patient was: *I don't know/I don't want to answer*

BEAT Project: Survey for Patients with Myeloproliferative Diseases

34. How much time does the journey from your house to the hospital usually takes?

- ◯ Less than 30 min
- ◯ Between 30 min and 1 hour
- ◯ Between 1 and hours
- ◯ More than 2 hours

35. Usually, in total, how much time do you spend on the following activities when you go to the hospital? If you don't perform an activity, select the corresponding answer

	Enter the number of minutes spent	
Blood collection		◯ I did not perform this activity
Check-up		◯ I did not perform this activity
Transfusion		◯ I did not perform this activity

36. Do you feel you have received **adequate information** regarding the following topics and associated issues:

	Not at all	A little	Enough	A lot	N/A
Myelofibrosis	◯	◯	◯	◯	◯
Anemia	◯	◯	◯	◯	◯
Transfusions	◯	◯	◯	◯	◯
Pharmacological treatments for myelofibrosis	◯	◯	◯	◯	◯

37. What have been your **sources of information** (possible multiple choices) on the following topics and associated issues:

	Hematologist	General Physician	Family / Acquaintances	Internet	Magazines	None	Patient Associations	N/A
Myelofibrosis	◯	◯	◯	◯	◯	◯	◯	◯
Anemia	◯	◯	◯	◯	◯	◯	◯	◯
Transfusions	◯	◯	◯	◯	◯	◯	◯	◯
Pharmacological treatments for myelofibrosis	◯	◯	◯	◯	◯	◯	◯	◯

38. Do you feel you have **understood the information** received regarding the following topics and associated issues:

	Not at all	A little	Enough	Completely	N/A
Myelofibrosis	◯	◯	◯	◯	◯
Anemia	◯	◯	◯	◯	◯
Transfusions	◯	◯	◯	◯	◯
Pharmacological treatments for myelofibrosis	◯	◯	◯	◯	◯

Where written N/A, the actual option shown to the patient was: *I don't know/I don't want to answer*

BEAT Project: Survey for Patients with Myeloproliferative Diseases

39. Do you usually ask the doctor questions to better understand your medical condition or to better understand how to manage therapies? ◯ Not at all ◯ A few ◯ Enough ◯ A lot ◯ N/A

40. Have you been provided with psychological support? ◯ Yes ◯ No ◯ N/A

41. If answered *«Yes»* to question 40: After how many months after the diagnosis of myelofibrosis? *after*______*months* ◯ Right away ◯ N/A

42. If answered *«Yes»* to question 40: Did you only pay the co-payment for psychological support? ◯ Yes ◯ No, I had to pay for it in full ◯ N/A

43. Do you participate in support groups? ◯ Yes ◯ No ◯ N/A

44. If answered *«Yes»* to question 43: How often do you participate in support groups? *every*______*days* *every*______*weeks* ◯ N/A

Where written N/A, the actual option shown to the patient was: *I don't know/I don't want to answer*
