# Supplementary material for: Anemia and blood transfusions in myelofibrosis: economic and organizational impact on Italian patients, caregivers and hospitals
Source: Front Oncol. 2025 Mar 7;15:1549023. doi: 10.3389/fonc.2025.1549023 (PMC11926708; doi:10.3389/fonc.2025.1549023)
Supplement: Supplementary file 1 [file DataSheet1.zip › Data Sheet 1/Supplementary Table 1.docx]

| **Data for the ‘average center’** | Data source | Method for calculating the average |
| --- | --- | --- |
| Average number of patients with MF managed annually | Clinicians | Simple average of the values reported by clinicians |
| % of patients by patient type | Clinicians | Weighted average, with values weighted by the number of patients treated at each clinician's center |
| Number of annual accesses by patient type | Clinicians | Simple average of the values reported by clinicians |
| Healthcare personnel time | Clinicians | Simple average of the values reported by clinicians |
| Waiting and activity times for patients | Clinicians | Simple average of the values reported by clinicians |
| Travel time home-hospital | Patients’ questionnaire | Simple average derived from total responses |
| Travel time home-laboratory for blood test only accesses | Assumption | Estimated at 15 minutes |
| % of accompanied patients | Clinicians | Weighted average, with values weighted by the number of patients treated at each clinician's center |
| Average number of caregivers accompanying patients | Patients’ questionnaire | Simple average derived from total responses |
| % of patients and caregivers employed | Clinicians | Weighted average, with values weighted by the number of patients treated at each clinician's center |
| Corporate cost of healthcare personnel | Literature / data research | Direct extraction from literature and data available |
| Average volume of blood transfused per session | Clinicians | Weighted average, with values weighted by the number of patients treated at each clinician's center |
| Average cost per blood bag | Literature / data research | Direct extraction from literature and data available |
| Structural costs (shared, general, administrative, amortization, non-health goods, and non-health services) | Literature / data research | Direct extraction from literature and data available |

**Supplementary Table 1. List of the average data and parameters used to define the ‘average center.’** Average values were calculated based on data obtained from clinicians’ interviews or extracted from patient questionnaire responses. In some instances, values were derived from literature or available datasets (e.g., average personnel salaries, average cost per blood bag), or estimated by the authors (i.e. travel time to laboratory for blood test-only accesses). The third column specifies the method used to calculate each average value. The formula applied for determining the weighted averages is illustrated in Supplementary Figure 1.
